# Prenatal Exposure to Bereavement and Type-2 Diabetes: A Danish Longitudinal Population Based Study

**DOI:** 10.1371/journal.pone.0043508

**Published:** 2012-08-28

**Authors:** Jiong Li, Jørn Olsen, Mogens Vestergaard, Carsten Obel, Jette Kolding Kristensen, Jasveer Virk

**Affiliations:** 1 Aarhus University, Aarhus, Denmark; 2 Research Unit for General Practice, Department of Public Health, Aarhus University, Aarhus, Denmark; 3 Department of Epidemiology, School of Public Health, University of California Los Angeles, Los Angeles, California, United States of America; Erasmus Medical Center, The Netherlands

## Abstract

**Background:**

The etiology of type-2 diabetes is only partly known, and a possible role of prenatal stress in programming offspring for insulin resistance has been suggested by animal models. Previously, we found an association between prenatal stress and type-1 diabetes. Here we examine the association between prenatal exposure to maternal bereavement during preconception and pregnancy and development of type-2 diabetes in the off-spring.

**Methods:**

We utilized data from the Danish Civil Registration System to identify singleton births in Denmark born January 1^st^ 1979 through December 31^st^ 2008 (N = 1,878,246), and linked them to their parents, grandparents, and siblings. We categorized children as exposed to bereavement during prenatal life if their mothers lost an elder child, husband or parent during the period from one year before conception to the child’s birth. We identified 45,302 children exposed to maternal bereavement; the remaining children were included in the unexposed cohort. The outcome of interest was diagnosis of type-2 diabetes. We estimated incidence rate ratios (IRRs) from birth using log-linear poisson regression models and used person-years as the offset variable. All models were adjusted for maternal residence, income, education, marital status, sibling order, calendar year, sex, and parents’ history of diabetes at the time of pregnancy.

**Results:**

We found children exposed to bereavement during their prenatal life were more likely to have a type-2 diabetes diagnosis later in life (aIRR: 1.31, 1.01–1.69). These findings were most pronounced when bereavement was caused by death of an elder child (aIRR: 1.51, 0.94–2.44). Results also indicated the second trimester of pregnancy to be the most sensitive period of bereavement exposure (aIRR:2.08, 1.15–3.76).

**Conclusions:**

Our data suggests that fetal exposure to maternal bereavement during preconception and the prenatal period may increase the risk for developing type-2 diabetes in childhood and young adulthood.

## Introduction

The etiology of type-2 diabetes is only partly known but is related to obesity, dietary and genetic factors [1]. A possible role of prenatal stress in programming offspring for insulin resistance has been considered [2] and studied in animal experimental models [3]. However, no data from human populations have been published. Prolonged exposure to severe stress may disrupt homeostasis, the body’s physiological response to stress [4], [5]. Accumulating evidence suggests that maternal stress during and before pregnancy affects the placenta, liver and pancreas [3]. Placental development begins very early in pregnancy with implantation at seven days post-conception. Poor implantation can lead to inadequate angiogenesis and inadequate substrate delivery to the fetus throughout the pregnancy [6]. Maternal stress can also alter the early intrauterine environment if it leads to poor nutrition, hypertension, chronic infections or inflammation. If normal implantation and placentation is interrupted by these events, epigenetic changes may program insulin resistance in the off-spring later in life.

The placental dehydrogenase enzyme 11β-HSD2 converts active glucocorticoids to inactive metabolites [7] and reduces the risk of elevated levels of glucocorticoids to the fetus [8], but this protection is not complete and does not appear until 20–24 weeks of gestation. If excess glucocorticoids reach the fetus at a critical stage of liver and pancreas development, these organs may be programmed for insulin resistance later [2], as part of the fetal ‘coping’ response to an adverse environment [9].

Previously, we showed that exposure to prenatal stress is associated with type-1 diabetes [10]. We hypothesize that exposure to stressful life events in mothers can affect proper implantation, alter the maturation of fetal organs and also lead to increased susceptibility to insulin resistance later in life. Bereavement was our indicator of stress since it almost uniformly induces severe stress, and has been shown to induce glucocorticoid production [11]–[13]. Death of a close relatives can be accurately ascertained using Danish Civil Registration System. The aim of this paper is to assess the risk of type-2 diabetes among children whose mothers were exposed to bereavement during the pre-natal or preconceptual period using our previously established Danish pregnancy cohort [10].

## Methods

This study was approved by the Institutional Review Board at the University of California at Los Angeles, the Danish Data Protection Agency (Ref no. 2008-41-2680) and the Ethics Committee for Central Jylland in Denmark (Ref no. M-20100252). Individual written consent was not obtained from study participants as there was no record linking the subjects to identifying information. Methods described in this paper have been described elsewhere [10]. In short, this study is a population-based follow-up study based on Danish national registers. We used data from the Danish Civil Registration System (CRS) to identify singleton births in Denmark born January 1^st^ 1979 through December 31^st^ 2008, N = 1,878,246 and linked them to their next of kin (mother, father, siblings, and mother’s parents). Since 1968, all live born children and new residents in Denmark are assigned a unique civil personal registration number, allowing for accurate linkage at the individual level between all national registries.

Information on birth outcomes, such as gestational age at birth and birthweight, were obtained from the Danish Medical Birth Registry which has been computerized since 1973. Annual information on maternal education, residence, and income was retrieved from the Fertility Database at Statistics Denmark, available since 1979. The date of conception was estimated by the date of birth minus gestational age, and the prenatal exposure period included from 12 months before conception until the birth of the child. We categorized children as exposed to bereavement during prenatal life if their mothers lost an elder child, husband or parent during the prenatal period; the remaining children were included in the unexposed cohort. All deaths were identified via the Death Registry which reports high validity on vital status [14]. Cohort members were followed from birth until first diagnosis of diabetes, death, emigration, or December 31^st^ 2008, whichever came first.

Diabetes in children and their parents was ascertained from the Diabetes and Hospital Registers. The National Hospital Register holds information on all discharges from Danish hospitals since 1977; outpatients have been included in the register since 1995. The Danish Diabetes Register was established in 2006 and also identifies individuals treated for diabetes outside of the hospital setting by combining data from the National Patient Register, the National Health Insurance Service Register and the Register of Medicinal Product Statistics [15]. Individuals with a diabetes diagnosis from the Diabetes Register that did not have a hospital diagnosis for type-1 diabetes were assumed to have type-2 diabetes. Diagnostic information was based on the Danish version of the International Classification of Disease (ICD) 8^th^ revision, from 1979 to 1993 (250) and 10^th^ revision, from 1994 onwards (E11).

We estimated incidence rate ratios (IRRs) from birth using log-linear poisson regression models and used person-years as the offset variable. The analysis was performed using PROC GENMOD in SAS version 9.1.3. Unadjusted and adjusted models were generated. Adjusted estimates of IRR (aIRR) included maternal age (≤18, 19–34, 35–40, 41+); residence (Copenhagen, cities with over 100,000 inhabitants, and other); income (1^st^, 2^nd^, 3^rd^, and 4^th^ quartile); maternal education (primary, secondary, high); parental diabetes status (any hospitalization diabetes ICD code); marital status (married, not married); sibling order (1,2,3,4+); calendar year (1979–1989, 1990–1999, 2000–2004, 2004–2008) and offspring’s sex (male, female). Age, residence, calendar period, maternal education, maternal income and parental marital status were treated as time-dependent variables.

To examine the association between timing of the bereavement and diabetes, we categorized exposed children by prenatal trimesters. To examine the relationship between type of bereavement and diabetes, we categorized exposed children by relationship to the deceased. We further categorized the cause of death by traumatic death (traumatic causes: ICD-8 codes 7950–7959, ICD-10 codes R95–R97; motor vehicle accidents: ICD-8 codes 8100–8230, ICD-10 codes V01–V89; suicide: ICD-8 codes 950–959, ICD-10 codes X60–X84; and other accidents and violence: ICD-8 codes 800–807, ICD-10 codes V90–V99, W00–X59, X85–Y89); and death from other causes.

**Table 1:** Background Statistics of all live Singleton Danish Births by Bereavement Status (1997–2008) ‡.

	Total Cohort (N = 1,878,246)		Exposed (N = 45,302)		Unexposed (N = 1,832,944)		P-Value
	N	(%)	N	(%)	N	(%)	
**Maternal age**							
≤18	21,652	1.2	350	0.8	21302	1.2	
19–34	1,628,883	86.7	37,723	83.3	1591160	86.8	
35–40	209,965	11.2	6,636	14.7	203329	11.1	
41+	17,746	0.9	593	1.3	17153	0.9	<.0001
**Paternal age**							
≤18	39,683	2.2	883	2.1	38,800	2.2	
19–34	1,365,866	76.1	37,076	72.6	1,328,790	76.1	
35–45	354,388	19.8	9,918	23.2	344,470	19.7	
46+	34,053	1.9	905	2.1	33,148	1.9	<.0001
**Household Income**							
1st quartile	71,346	3.8	911	2.0	70,435	3.8	
2nd quartile	473,109	25.2	10,234	22.6	462,875	25.3	
3rd quartile	769,339	41	19,781	43.7	749,558	40.9	
4th quartile	564,435	30.1	14,375	31.7	550,060	30.0	<.0001
**Maternal Education**							
Primary	605,514	33.1	14,342	31.7	591,172	32.3	
Secondary	578,708	31.7	14,715	32.5	563,993	30.8	
High	643,633	35.2	15,777	34.8	627,856	34.3	<.0001
**Marital status**							
Not married	736,521	39.2	18,904	41.7	717,617	39.2	
Married	1,141,722	60.8	26,398	58.3	1,115,324	60.9	<.0001
**Child's sex**							
Male	963,648	51.3	23,116	51.0	940,532	51.3	
Female	914,598	48.7	22,186	49.0	892,412	48.7	0.2286
**Maternal Residence**							
Copenhagen	404,440	21.5	9,247	21.6	395,193	20.4	
Big cities*	186,714	9.9	4,419	10.0	182,295	9.8	
Other	1,287,092	68.5	31,636	68.5	1,255,456	69.8	<.0001
**Birth Weight (grams)**							
<1500	56,497	3.0	1,181	2.6	55,316	3.0	
1500–2500	80,928	4.3	2,321	5.1	78,607	4.3	
>2500	1,740,821	92.7	41,800	92.3	1,699,021	92.7	<.0001
**Gestational age (weeks)**							
<34	91,198	4.9	1,613	3.6	89,585	4.9	
34–36	82,475	4.4	2,371	5.2	80,104	4.4	
>37	1,704,573	90.8	41,318	91.2	1,663,255	90.7	<.0001

‡ Maternal age, paternal age, household income, maternal education, marital status, and maternal residence obtained from the time of birth.

* Cities with over 100,000 residents.

## Results

Children in our study were followed up from 2 to 32 years, and the mean length of follow-up was 16.4 years. Exposed and unexposed children were comparable on most characteristics at baseline but as expected mothers of bereaved children were slightly older and had more children (see Table 1). We identified 45,302 children exposed to bereavement during or before their prenatal life. There were 28,199; 5,627; 5,809; and 5,823 children exposed during preconception, first, second, and third trimesters of pregnancy, respectively. Furthermore, there were 514; 6,813 and 38,131 children exposed to death of a father, sibling and maternal grandparent, respectively. The average age of onset in this cohort for type-2 diabetes was 18.1 and 20.1 years for boys and girls, respectively. Table 2 provides adjusted and unadjusted incidence rate ratios for the association between maternal bereavement and type-2 diabetes by relationship to the deceased. We found that children exposed to bereavement during their prenatal life were more likely to have a type-2 diabetes diagnosis later in life (aIRR:1.31, 1.01–1.69). These findings were most pronounced when bereavement was caused by traumatic death (aIRR: 1.95, 1.17–3.24). We also found that the second trimester may be the most sensitive period of exposure (aIRR:2.08, 1.15–3.76), see Table 3.

**Table 2:** Incidence rate ratios for Type-2 Diabetes by relationship to deceased (1979–2008)*.

Exposed to death of:	Exposed Cases	IRR	aIRR	Lower CL	Upper CL
Father	1	–	–	–	–
Sibling	17	1.87	1.51	0.94	2.44
Father/Sibling	18	1.84	1.50	0.94	2.39
Grand parent	41	1.00	1.24	0.91	1.69
Father/sibling/grandparent	59	1.16	1.31	1.01	1.69

* Adjusted for maternal residence, income, education, marital status, sibling order, calendar year, sex, parents’ history of diabetes.

## Discussion

We found evidence to suggest that fetuses exposed to maternal bereavement during the preconception and the prenatal period are at increased risk for developing type-2 diabetes. Chance should be considered as a possible explanation since the number of exposed cases in our cohort is small. Bereavement is a complex exposure of which severe stress is an important component; other components potentially influencing pregnancy outcomes include anxiety, use of medication (including illicit drugs), loss of sleep or appetite. Social support may also influence coping mechanisms and thus the biological response to stress. Previously, we reported a similar association for type-1 diabetes [10] which supports that severe stress may interfere with the development of Langerhan cell function during pregnancy. Our findings combined with the increasing incidence of both types of diabetes indicate that these diseases may share common environmental causes.

**Table 3:** Incidence rate ratios for Type-2 Diabetes by Timing of Bereavement* and Cause of Death‡ (1979–2008).

Timing of exposure	ExposedCases	IRR	aIRR	LowerCL	UpperCL
**Preconception**					
Traumatic exposure	8	1.24	1.52	0.76	3.05
Non-Traumatic exposure	25	0.98	1.04	0.71	1.54
Any exposure	33	1.03	1.13	0.81	1.59
**1st Trimester (0**–**12)**					
Traumatic exposure	2	–	–	–	–
Non-Traumatic exposure	5	0.60	0.68	0.22	2.11
Any exposure	7	0.94	1.11	0.50	2.48
**2nd Trimester(13–27)**					
Traumatic exposure	3	–	–	–	–
Non-Traumatic exposure	7	1.57	1.85	0.92	3.70
Any exposure	10	1.69	2.08	1.15	3.76
**3rd Trimester (28–42)**					
Traumatic exposure	1	–	–	–	–
Non-Traumatic exposure	9	1.73	1.97	1.02	3.79
Any exposure	10	1.52	1.81	0.97	3.37

* Adjusted for maternal residence, income, education, marital status, sibling order, calendar year, sex, parents’ history of diabetes.

‡ Traumatic death includes deaths from motor vehicle accidents, suicide, other accidents and acts of violence.

The developmental origins of health and disease theory proposes several mechanisms by which insulin production may be programmed in early life, including exposure to excess glucocorticoid hormones [3], [16], [17]. In short, fetal programming of diabetes may occur via exposure of the fetus to excess glucocorticoid hormones which may be higher in mothers exposed to stress during or before pregnancy. We observed the second trimester (27 weeks) to be sensitive to bereavement, perhaps because the placental dehydrogenase enzyme 11β-HSD2 which converts active glucocorticoids to inactive metabolites [7] is not protective until 20–24 weeks of gestation.

Previous reviews and other studies [18], [19] have discussed possible mechanisms of how the effects of prenatal stress can be emitted prenatally from mother to child, such as through transplacental maternal stress hormones (cortisol) to the fetus; maternal stress-induced release of placental hormones (corticotrophin-releasing hormone) that enter the fetal circulation; and maternal stress-induced alterations in uteroplacental blood flow. Changes in the uteroplacental blood flow produced by maternal stress hormones may restrict nutrients and thus program the fetus to develop insulin resistance. Furthermore, cortisol, a catabolic hormone, induces several processes leading to increased concentrations of blood glucose [7]. Therefore, it’s possible that the hormonal changes in the fetal circulation induced by maternal stress elevate levels of glucose in maternal and that fetal circulation can contribute to the development of insulin resistance. Experiments in animal models and one human study showed that insulin resistance and other manifestations of the metabolic syndrome can be induced by manipulating maternal nutrition or exposing the mother to synthetic glucocorticoids or prenatal stress [20]–[25].

Age and sex specific prevalence among adult Danes with type-2 diabetes has been previously reported [26]. The main strengths of our study are the large cohort size and high quality of data. Information in the CRS has been made available for research purposes by Danish legislation. Parental links for individuals born in Denmark since 1969 are considered accurate; Danish legislation requires all legal address changes be submitted to the CRS [27]. The main limitation of our study is the lack of inclusion of other sources of stressors, baseline measures of biological stress responses and measures of allostatic load. Longitudinal biological sampling is difficult to obtain due to ethical and economic reasons. Though despite these shortcomings we believe the exposure contrast between the exposed and unexposed groups is substantial.

While one cannot avoid bereavement as a potential stressor during pregnancy one may be able to reduce the stress exposure that follows, avoid other sources of stress, or mitigate the effects via therapy or support. This study suggests that exposure to severe stress during the period surrounding pregnancy may have life-long effects on the offspring. We did not find a strong association between bereavement and diabetes when cause of death was non-traumatic or that of a grandparent, suggesting that only the most acute stress exposures have a programming effect on this disease during this follow-up time. Furthermore, if this association reflects a causal link to cortisol exposure, prenatally prescribed medications may have a similar effect.

This is the largest population sample of bereavement and diabetes ever to be studied. Previously, a small study (obs = 58) reported an association between markers of prenatal psychosocial stress and glucose metabolism, suggesting a link between stress and diabetes [28]. The findings of this study may have public health implications especially in settings where sources of intense stress are increasing, such as in areas of political unrest, conflict or environmental distress. Replication of these findings is of interest particularly in large cohorts or among populations where acute stressors can be identified, such as in emergency situations. Prenatal stress exposure, hormonal or other stress like exposure, may be one of the contributing factors increasing the incidence of both type-1 and type-2 diabetes.
