# Correction: Prenatal Exposure to Bereavement and Type-2 Diabetes: A Danish Longitudinal Population Based Study

**DOI:** 10.1371/annotation/dbd21894-4722-499c-afaf-b03015fae7d8

**Published:** 2013-08-15

**Authors:** Jiong Li, Jørn Olsen, Mogens Vestergaard, Carsten Obel, Jette Kolding Kristensen, Jasveer Virk

There was an error in the order of authors in the paper byline. The correct order is: Jasveer Virk, Jiong Li, Mogens Vestergaard, Carsten Obel, Jette Kolding Kristensen, Jørn Olsen

The correct citation is: Virk J, Li J, Vestergaard M, Obel C, Kristensen JK, et al. (2012) Prenatal Exposure to Bereavement and Type-2 Diabetes: A Danish Longitudinal Population Based Study. PLoS ONE 7(8): e43508. doi:10.1371/journal.pone.0043508 

